# Compensation claims after hip arthroplasty surgery in Norway 2008–2018

**DOI:** 10.1080/17453674.2021.1872901

**Published:** 2021-01-18

**Authors:** Tommy Frøseth Aae, Rune Bruhn Jakobsen, Ida Rashida Khan Bukholm, Anne Marie Fenstad, Ove Furnes, Per-Henrik Randsborg

**Affiliations:** a Department of Orthopaedic Surgery, Health Møre and Romsdal HF, Kristiansund Hospital, Kristiansund;; b Department of Orthopaedic Surgery, Akershus University Hospital, Lørenskog;; c Department of Health Management and Health Economics, Institute of Health and Society, The Medical Faculty, University of Oslo;; d Norwegian System of Compensation to Patients, Oslo;; e The Norwegian Arthroplasty Register, Department of Orthopaedic Surgery, Haukeland University Hospital, Bergen;; f Department of Clinical Medicine, Faculty of Medicine, University of Bergen, Bergen, Norway;; g Sports Medicine Institute, Hospital for Special Surgery, New York, USA.

## Abstract

Background and purpose — Orthopedic surgery is one of the specialties with most compensation claims, therefore we assessed the most common reasons for complaints following total hip arthroplasty (THA) reported to the Norwegian System of Patient Injury Compensation (NPE) and viewed these complaints in light of the data from the Norwegian Arthroplasty Register (NAR).

Patients and methods — We collected data from NPE and NAR for the study period (2008–2018), including age, sex, and type of complaint, and reason for accepted claims from NPE, and the number of arthroplasty surgeries from NAR. The institutions were grouped by quartiles into quarters according to annual procedure volume, and the effect of hospital procedure volume on the risk for accepted claim was estimated.

Results — 70,327 THAs were reported to NAR. NPE handled 1,350 claims, corresponding to 1.9% of all reported THAs. 595 (44%) claims were accepted, representing 0.8% of all THAs. Hospital-acquired infection was the most common reason for accepted claims (34%), followed by wrong implant position in 11% of patients. Low annual volume institutions (less than 93 THAs per year) had a statistically significant 1.6 times higher proportion of accepted claims compared with higher volume institutions.

Interpretation — The 0.8% risk of accepted claims following THAs is 1.6 times higher for patients treated in low-volume institutions, which should consider increasing the volume of THAs or referring these patients to higher volume institutions.

In Norway, compensation claims are handled by the Norwegian System of Patient Injury Compensation (NPE) and not by the judiciary system. If a patient in Norway suffers a complication due to a treatment error, within either the public or private healthcare sector, the patient can file a free-of-charge compensation claim to NPE. For claims to be accepted, 3 criteria must be met. 1st, the injury must have occurred during medical treatment (examination, diagnosis, or treatment/lack of treatment) or during follow-up, and the treatment must be deemed substandard or erroneous based on current treatment guidelines. 2nd, the injury must have led to financial loss (currently set at €1,000) or to a persistent medical impairment of minimum 15%. Lastly, the claim must be filed within 3 years after the patient became aware that the injury was likely a treatment error. There is 1 exception clause to these criteria: If the injury is rare and severe, claims may be accepted even when no treatment error has been identified. The amount of compensation is being reviewed on an individual basis and calculated to cover the patient’s loss of income and increased medical expenses due to the treatment injury.

Orthopedic surgery is one of the specialties with most compensation claims following medical treatment (Jena et al. 2011). Previous studies on compensation claims after THAs have been limited by methodological inadequacies, such as short study period or limited sample size with claims ranging from 40 to just above 300 (Bhutta et al. 2011, Bokshan et al. 2017, Novi et al. 2020). We evaluated claims following both primary and revision THAs filed at the NPE from 2008 to 2018 and compared these findings with data from NAR, with a focus on institutional procedure volume.

## Patients and methods

### Patients

All patients of any age who filed a claim with NPE following primary or revision THA from 2008 to 2018 were included. Patients who underwent primary hemiarthroplasty or THA for a femoral neck fracture were excluded.

### Methods

The Norwegian Arthroplasty Register (NAR) was founded in 1987, with aims to monitor the safety and epidemiology of total joint arthroplasties (Havelin et al. 2000). Annually, approximately 98% of primary THAs and 93% of revision THAs are reported to NAR (NAR 2020).

Data from NAR was collected for the study period (2008 through 2018). The data was stratified by the number of arthroplasty procedures performed every year per institution. The institutions were then divided by quartiles into quarters according to annual procedure volume groups: Quarter 1 (Q1) consisted of 7 institutions with an annual volume of less than 93 hip arthroplasties. Quarter 2 (Q2) included 8 institutions that performed 93–263 procedures per year. The third quarter (Q3) comprised 8 institutions with an annual surgical volume of 264–466 hips, and, finally, the highest volume quarter (Q4) included 7 institutions that perform more than 466 hip arthroplasties yearly.

All claims filed at the NPE following THA in the study period were collected, both primary THA and revision THA. The data was stratified by institution, the patient’s age and sex, type of complication, any reoperations, and any fatalities. The reasons for the claims were recorded, together with the decision made by NPE (accepted or rejected claims). When evaluating the outcome of claims on institutional volume, the outcome of interest was the proportion of procedures resulting in an accepted claim, with the individual institution as the analysis unit.

### Statistics

Continuous variables were described by mean (SD) or median (range) while categorical data were presented in frequencies. Groups were compared using the chi-square test. The institutions by procedure volume were compared using ANOVA after asserting conditions were met, and p-values adjusted for multiple testing by Tukey’s comparison test. Between-quarter associations were quantified by odds ratio. A p-value < 0.05 was considered statistically significant. The data was analyzed using IBM SPSS version 26 (IBM Corp, Armonk, NY, USA).

### Ethics, funding, and conflicts of interest

The Regional Ethical Committee deemed approval not necessary as all data is based on already anonymized records (REK 15.10.10). The study was funded by research grants from the Norwegian Research Council (the Norwegian Cartilage Project, grant number 2015107). The authors declare no conflict of interests.

## Results

During the study period from 2008 to 2018 (11 years) 70,327 hip arthroplasties were reported to NAR, of which 86% were primary procedures and 14% were revision THAs. During this period, NPE received 53,000 claims, of which 31% were related to orthopedic surgery and 1,350 claims were filed following hip arthroplasties, representing 1.9% of all hip procedures reported to NAR. Patients filing a claim with NPE were younger than the average age of patients reported to NAR (64 years, SD 12) compared with 68 (SD 11) years, p < 0.001). NAR received reports on 65% of women, which was comparable to the 67% of claims that were put forward by women (p = 0.2) (Table 1).

**Table 1.** Demography of hip arthroplasty procedures reported to the Norwegian Arthroplasty Registry (NAR) and claims due to treatment injuries following hip arthroplasties filed with the Norwegian System of Patient Injury Compensation (NPE) during 2008–2018

Factor	Hip procedures reported to NAR n = 70,327	Compensation claims filed to NPE n = 1,350	Accepted claims n = 595 (44%)	Rejected claims n = 755 (56%)
Age, mean (SD)	68 (11)	64 (11)	64 (11)	64 (12)
range	11–100	20–89	21–89	20–89
Females, n (%)	45,572 (65)	899 (67)	348 (59)	551 (73)

SD, standard deviation.

595 (44%) of 1,350 claims were accepted, representing 0.8% of all hip arthroplasties reported to NAR in the study period. 549 claims were accepted following primary procedures (0.9% of all primary hip arthroplasties) and 46 claims were accepted after revision THA (0.5% of all revisions) (p < 0.001).

Hospital-acquired infection was the most common reason for accepted claims (34%), followed by wrong implant position (11%) (Table 2). There was a decline in claims due to abductor deficiency towards the end of the period, with 67 claims (10 granted) between 2011 and 2014 compared with 20 claims (4 granted) during 2015–2018 (p = 0.04).

**Table 2.** Reasons for claims (n = 595) accepted by the Norwegian System of Patient Injury Compensation for treatment injuries following total hip arthroplasty during 2008–2018

Reason for accepted claims	n (%)
Hospital-acquired infection	201 (34)
Malposition of implant	67 (11)
Treatment failure	50 (8.4)
Anisomelia	50 (8.5)
Nerve injury	45 (7.6)
Aseptic loosening **^a^**	35 (5.9)
Abductor deficiency	33 (5.5)
Exception clause	30 (5.0)
Perioperative fracture	21 (3.5)
Technical error	17 (2.9)
Delayed treatment	16 (2.7)
Wrong indication	11 (1.8)
Pain	7 (1.2)
Component failure	4 (0.7)
Delayed diagnosis	3 (0.5)
Artery injury	2 (0.3)
Lack of information	2 (0.3)
Anesthesia	1 (0.2)

**^a^** Within 3 years.

17 of 23 claims from private hospitals were accepted, compared with 578 of 1,327 claims from public hospitals (p = 0.004).

5 claims involving fatalities were filed, all occurring in male patients, and all 5 claims were accepted. 3 patients aged 63, 66, and 88 succumbed to complications related to surgical site infection following a primary THA. A 67-year-old patient with known diabetes mellitus underwent revision surgery and died due to hypoglycemia as blood glucose was not followed up according to guidelines. A 52-year-old patient suffered a pulmonary embolus following a primary THA despite prophylactic administration of low-molecular-weight heparin. No treatment error was identified in that case, but compensation was granted based on the exception clause.

At the time of writing [August, 2020], 90% of accepted claims have had the compensation calculated, amounting to €15.4 million that has been paid out in compensation following hip arthroplasties performed during the period 2008–2018, with average compensation of €26,000.

### Institutional procedure volume

Institutions with the lowest annual volume (< 93, Q1) had a statistically significant higher fraction of accepted claims per procedure compared with higher volume institutions (Figure). The odds ratio for a claim to be accepted following a hip arthroplasty performed at a low-volume institution was 1.6 (95% CI 1.1–2.5) compared with higher volume institutions (Table 3).

**Figure:**
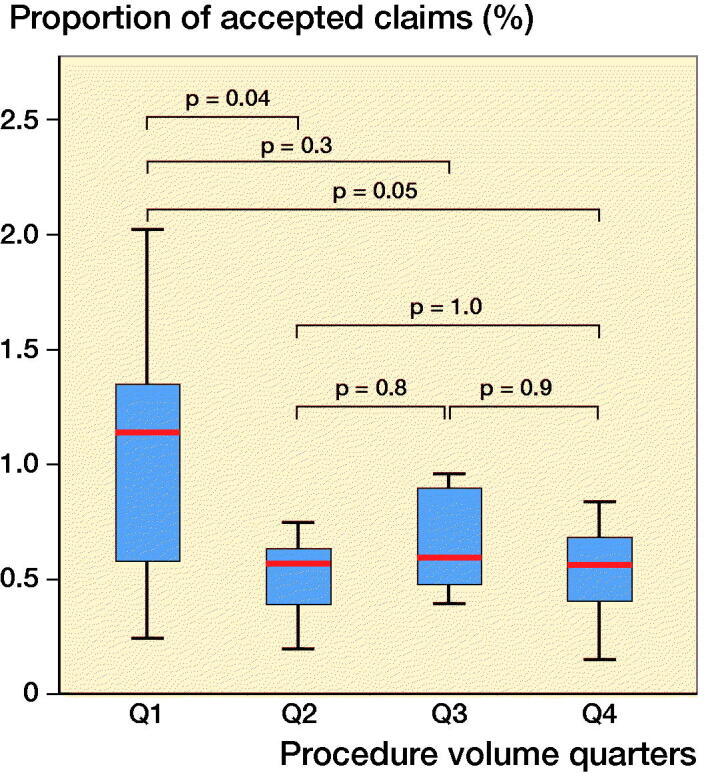
Proportion of accepted claims by number of surgeries stratified by annual hospital procedure volume. The 4 categories represent quarters, see Table 3. P-values derived from ANOVA adjusted with Tukey’s comparison test.

**Table 3.** Likelihood of accepted claims from the Norwegian System of Patient Injury Compensation during 2008–2018 by annual procedure volume divided by quartiles into quarters

Quarter (Q)	Odds ratio (95% CI)
Q1 vs. all other	1.6 (1.1–2.5)
Q1 vs. Q2	1.8 (1.1–3.0)
Q1 vs. Q3	1.4 (0.9–2.2)
Q1 vs. Q4	1.7 (1.1–2.7)
Q2 vs. Q3	0.8 (0.6–1.0)
Q2 vs. Q4	0.9 (0.7–1.2)
Q3 vs. Q4	1.2 (1.0–1.5)

Q1, Quarter 1 (< 93 annual procedures);

Q2, Quarter 2 (93–263 annual procedures);

Q3, Quarter 3 (264–466 annual procedures);

Q4, Quarter 4 (> 466 annual procedures).

CI, confidence interval.

## Discussion

The main finding of the present study is that approximately 1% of all patients undergoing THA between 2008 and 2018 were granted compensation by NPE. The probability of being granted compensation claims due to a treatment error is 1.6 times more likely if the procedure was performed in a low-volume institution compared with institutions with higher volumes of THAs. We found a 1.6 times increased risk of suffering a treatment error that led to accepted claims by NPE if the THA was performed in the lowest volume institutions compared with all other institutions. However, the proportion of accepted claims was not statistically significantly different between the lowest volume institutions and the 2nd highest volume institutions. The 2nd highest quarter consists of 8 institutions, including 4 large university clinics in Norway. These institutions typically treat high-risk patients and complex primary and revision cases, whereas the highest volume institutions consist of specialized “production” institutions that treat a high volume of relatively low-risk patients. We believe this might explain why Q3 has a somewhat higher number of accepted claims, despite relatively large annual procedure volumes.

Low institutional procedure volumes increase the risk of adverse outcomes following elective hip arthroplasty and revision surgery (Glassou et al. 2016, Mufarrih et al. 2019). Our study confirms that low-volume institutions also have a higher ratio of accepted claims compared with institutions with higher volumes of THAs. However, the risk of ending up with compensation after a hip arthroplasty is only moderately elevated for the lowest volume institutions, with an odds ratio of 1.6 and a 95% confidence interval approaching 1.0. In contrast, in a similar study on compensation claims following total knee arthroplasty (TKA), we found a 3-fold increased risk of accepted claims in the lowest volume institutions (Randsborg et al. 2020). The effect of institutional volume on accepted claims seems to be much more pronounced for TKAs than for THAs as this is also found in a Finnish report (Järvelin et al. 2012). This is not surprising, since hospital volume has a greater effect on adverse outcome following knee arthroplasties compared with hip arthroplasties (Katz et al. 2004, Shervin et al. 2007). THA is indisputable a more successful procedure with higher patient satisfaction and less complications compared to TKA (Bourne et al. 2010), which may explain why institutional volume seems to have less impact on compensation after hip arthroplasties than after knee arthroplasties.

The dominant reason for accepted claims was hospital-acquired infection, accounting for one-third of accepted claims. Hospital-acquired infection of an arthroplasty leads to prolonged hospitalization, and increased morbidity and mortality, with extensive costs to the society (Senard et al. 2019). This is a reminder that all parties involved in arthroplasty surgery should strive to reduce the risk of infection.

The exception clause grants compensation to patients who suffer from infection following joint replacement, even when no treatment error has been identified. A similar decision has been made for early (within 3 years) aseptic loosening. It is a pragmatic policy in a no-blame compensation system. However, not all infections will automatically lead to compensation. All claims are reviewed independently. Patients with comorbidity, high infection risk, and poor compliance may not be granted compensation if infection occur.

Other leading causes for accepted claims were wrong implant position, treatment failure (no further explanation was given), anisomelia, and nerve injury.

Abductor deficiency was registered in 6% of accepted claims. This is likely related to the surgical technique and the direct lateral approach to the hip joint (Amlie et al. 2014, Winther et al. 2016). This approach has decreased substantially in comparison with posterior and anterior approaches in Norway in recent years and is now performed in less than 5% of all THAs (NAR 2020). We believe this explains the decline in claims due to abductor deficiency registered at the end of the study period.

Pain was a common reason for claim (Table 1). NPE registered 215 claims due to pain, although only 7 (3%) were accepted for compensation; pain alone does not serve as a cause for compensation, which is in line with previous reports from NPE (Clementsen et al. 2018, Randsborg et al. 2018, Aae et al. 2020).

Khan et al. (2020) assessed compensation claims based on the Danish arthroplasty register and found that 2.5% of all THAs filed a claim, which is somewhat higher than our findings of 1.9%. They reported nerve damage and insufficient or incorrect treatment as the leading causes for accepted claims. Half of the claims were accepted, which is similar to the 44% of claims accepted in our study. In contrast to our study, they did not include revision surgery. Our study also adds knowledge on accepted claims based on institutional volumes of THA, a topic that has received little attention in the literature.

Bhutta et al. (2011) reviewed compensation claims after hip and knee arthroplasties over a 5-year period in the United Kingdom. THAs due to trauma and revision surgery were excluded. They identified 271 claims that had reached a conclusion, where 109 (40%) resulted in payouts. Nerve injury, surgeon error, and pain were the 3 most common causes for claims. Our material contains 5 times as many claims as Bhutta et al. and includes revision surgery. Albeit nerve injury and surgeon error are common reasons for accepted claims in both studies, infection was far more common in our study. This discrepancy is likely caused by the exception clause in the Scandinavian compensation systems that grants compensation after infection even if no treatment error is identified. This is supported by a study on patient claims following prosthetic hip infections in Sweden, which found that 329 of 441 (75%) of claims were accepted (Kasina et al. 2018), which is the same as the 209 of 275 (76%) of infections that were accepted in our study.

2 studies from the United States analyzed malpractice lawsuits after THA and both identified substantially fewer claims than our study, which may relate to the systems (Bokshan et al. 2017, Samuel et al. 2019). In Norway, filing a complaint for compensation is free of charge and easily done online. Additionally, the system is a no-fault system with claims not directed at individual surgeons or institutions, and surgeons are required by law in case of complications to inform patients how to apply for compensation. In the United States, filing a complaint will normally require legal representation, which might constitute a higher threshold for filing a claim.

Some treatment errors found in our study are avoidable or at least modifiable with adjustments in medical practice. This is coherent with findings from Sweden, which stated that 49% of THA patients suffered an adverse advent that could have been prevented (Magnéli et al. 2020). Delayed diagnosis or treatment and erroneous indications have previously been identified as avoidable causes for compensation (Clementsen et al. 2018, Randsborg et al. 2018, Aae et al. 2020). During the study period, all hospitals in Norway implemented the use of the safe surgery protocol initiated by the World Health Organization (WHO 2020). No cases of wrong-sided surgery were identified in our study and this may relate to the implementation of these guidelines.

1 accepted claim was not due to the surgery itself, but to anesthesia and is a reminder that arthroplasty carries risks not directly related to the surgery.

This study has limitations. Any mislabeling of procedures may lead to some patients not being included in the study. NPE does not cover all possible complications following THA, and it is likely that some patients have experienced a complication due to a treatment error that would lead to compensation, but never filed a claim. These factors may induce biases to the database used in this study.

The data originates from 1 country using the principle of no-blame, which may reduce the generalizability of the study. However, the purpose of this study is to evaluate compensation claims to identify areas for potential improvement in our patient care, which is likely of universal interest.

In conclusion, the main reasons for compensation were a hospital-acquired infection and malposition of the implant. The findings suggest that small-volume institutions should consider increasing the volume of THAs or referring these patients to higher volume institutions. Several complications identified are reducible with adjustments to current clinical practice.
